# Congenital nephrotic syndrome

**DOI:** 10.1007/s00467-007-0633-9

**Published:** 2007-10-30

**Authors:** Hannu Jalanko

**Affiliations:** Hospital for Children and Adolescents, University of Helsinki, Stenbackinkatu 11, Helsinki, 00029 HYKS Finland

**Keywords:** Proteinuria, Nephrotic syndrome, Nephrin, Podocin, Podocyte, Kidney transplantation

## Abstract

Congenital nephrotic syndrome (CNS) is a rare kidney disorder characterized by heavy proteinuria, hypoproteinemia, and edema starting soon after birth. The majority of cases are caused by genetic defects in the components of the glomerular filtration barrier, especially nephrin and podocin. CNS may also be a part of a more generalized syndrome or caused by a perinatal infection. Immunosuppressive medication is not helpful in the genetic forms of CNS, and kidney transplantation is the only curative therapy. Before the operation, management of these infants largely depends on the magnitude of proteinuria. In severe cases, daily albumin infusions are required to prevent life-threatening edema. The therapy also includes hypercaloric diet, thyroxin and mineral substitution, prevention of thrombotic episodes, and prompt management of infectious complications. The outcome of CNS patients without major extrarenal manifestations is comparable with other patient groups after kidney transplantation.

## Introduction

Congenital nephrotic syndrome (CNS) is defined as heavy proteinuria starting within three months after birth. Nephrotic syndrome (NS) appearing later during the first year (4–12 months) is defined infantile, and NS manifesting thereafter is called childhood NS [1, 2]. These definitions have been used for decades in order to help the clinical diagnosis, although recent findings indicate that NS caused by a particular gene defect can manifest at various ages, questioning the rationale of the classification. Since the overall etiology, clinical features and management of CNS, however, are different from the more common forms of childhood NS, the terminology still seems warranted (Table 1).

**Table 1:** The etiology of congenital nephrotic syndrome (CNS)

Primary CNS
Nephrin gene mutations [*NPHS1*, Finnish type of CNS (CNF)]
Podocin gene mutations (*NPHS2*)
*WT1* gene mutations (Denys-Drash, isolated CNS)
*LamB2* gene mutations (Pierson syndrome, isolated CNS)
*PLCE1* gene mutations
*LMX1B* mutations (nail-patella syndrome)
*LamB3* gene mutations (Herlitz junctional epidermolysis bullosa)
Mitochondrial myopathies
CNS with or without brain and other malformations (no gene defect identified as yet)
Secondary CNS
Congenital syphilis
Toxoplasmosis, malaria
Cytomegalovirus, rubella, hepatitis B, HIV
Maternal systemic lupus erythematosus
Neonatal autoantibodies against neutral endopeptidase
Maternal steroid–chlorpheniramine treatment

## Glomerular filtration barrier

**Fig. 1:**
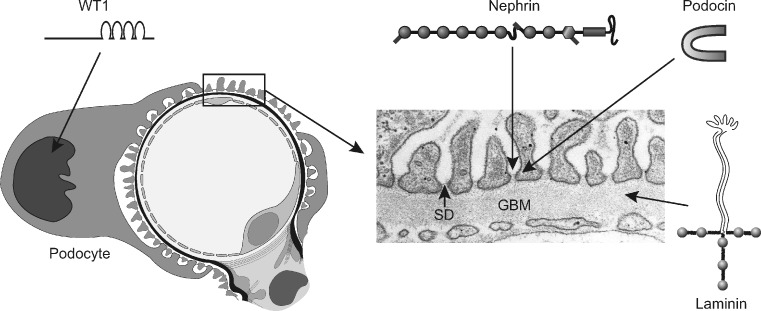
Cross-section of a glomerular capillary (*left*) and electron microscopy image of a normal capillary wall (*right*). *WT1* is a transcription factor important for podocyte function. *Nephrin* is a major component of the slit diaphragm (*SD*) connecting podocyte foot processes. *Podocin* is an adapter protein located intracellularly in the SD area. *Laminin* is a major structural protein of the glomerular basement membrane (*GBM*). Genetic mutations in these proteins lead to congenital nephrotic syndrome

The cardinal feature of CNS is the extensive leakage of plasma proteins into urine. In most cases, this is caused by mutations in genes encoding for structural or regulatory proteins of the kidney filtration barrier located in the glomerular capillary wall [3–5]. This filter is composed of three layers: fenestrated endothelium, glomerular basement membrane (GBM), and epithelial cell (podocyte) layer with distal foot processes and interposed slit diaphragms (SD) (Fig. 1). The barrier is an effective size- and charge-selective molecular sieve, and normally only water and small plasma solutes pass through it. The flux of albumin and larger plasma proteins is restricted by the GBM and especially SD, so that the protein content of the ultrafiltrate (primary urine) reaching the Bowman space is very low. The GBM’s role in glomerular permselectivity has been debated recently, but it is now known that proteinuria can be caused by a primary defect in either SD or GBM.

The GBM is a well-known protein network formed by type IV collagen, laminin, nidogen, and negatively charged proteoglycans. On the other hand, the precise molecular structure of the SD is still unresolved. Recently identified podocyte proteins, such as nephrin, Neph1, Neph2, FAT1, FAT2, and dendrin probably form the backbone of SD [6–8]. These proteins associate with each other extracellularly and interact with the adapter proteins, such as podocin, CD2AP, ZO-1, CASK, and MAGI-1, localized in the cytosolic part of the podocyte. These connect SD with the actin cytoskeleton of the podocyte foot process and take part in signal transduction from the slit diaphragm area into podocyte [9]. Actin network and the interacting proteins, such as α-actinin-4, are critical for maintenance of the complex structure of the podocyte. Interestingly, this molecular organization becomes disrupted in CNS and other proteinuric disorders, leading to effacement of podocyte foot processes.

## Different forms of CNS

### Nephrin gene (*NPHS1*) mutations

Mutations in *NPHS1* gene cause CNS, which was originally named as the Finnish type of CNS, or CNF [10]. Since CNF originally referred to a clinical entity (severe form of CNS), an abbreviation of NPHS1 (nephrotic syndrome type 1) was introduced to specify those cases known to be caused by *NPHS1* mutations [10]. In practice, both terms are used for the same disorder. The incidence of NPHS1 is one in 8,000 live births in Finland, and about half of the published cases are Finns. Currently, close to 100 mutations in the *NPHS1* gene have been identified [11–13]. Most patients have individual mutations of different types, except in Finland, where two founder mutations (Fin-major and Fin-minor) are detected in almost all cases. *NPHS1* codes for nephrin, which is a 1241-residue transmembrane adhesion protein of the immunoglobulin superfamily (Fig. 1). Nephrin is synthesized almost exclusively by glomerular podocytes and is a crucial component of the SD, as noted above.

Compared with many other genetic disorders, NPHS1 shows relatively little phenotypic variation [14]. Most of these children are born prematurely, with a birth weight ranging between 1,500 and 3,500 g. The placental weight is over 25% of the newborn weight in practically all cases. Amniotic fluid may be meconium stained, but the infants do not usually have major respiratory problems. Importantly, NPHS1 infants do not have extrarenal malformations. On the other hand, minor functional disorders, such as muscular hypotonia and cardiac hypertrophy, are common during the nephrotic stage. Proteinuria begins in utero and is detectable in the first urine sample after birth. Microscopic hematuria and normal creatinine values during the first months are typical. Heavy protein loss (up to 100 g/L) results in oliguria and severe edema if not treated. Hyperlipidemia is also present, as in other forms of NS.

The NPHS1 kidneys are large and in ultrasound scans cortical echogenicity is increased, and the corticomedullary border is indistinct. In renal histology, no single histological finding is pathognomonic for NPHS1. Expansion of glomerular mesangium and dilations in the proximal and distal tubules are the most characteristic findings. Interstitial fibrosis and inflammatory infiltrates, especially around the glomeruli, increase with time. Effacement of podocyte foot processes and disappearance of the filamentous image of SD are seen in electron microscopy [14].

### Podocin gene (*NPHS2*) mutations

Mutations in the *NPHS2* gene, encoding for a podocyte protein podocin, are a common cause of childhood (steroid-resistant) NS, but they are also important in the development of CNS [11–16]. In a recent report, *NPHS2* mutations accounted for half of the CNS cases in 80 European families, while *NPHS1* mutations were responsible for only one third of the cases [17]. The *NPHS2* mutations have also been found in CNS patients from Japan and elsewhere. They are typically “severe”, leading to nonfunctional podocin protein (often truncated). Since podocin is a podocyte-adapter protein required for proper targeting of nephrin into SD, nephrin expression also may be distorted in CNS caused by *NPHS2* mutations (Fig. 1) [18]. Coexistence of *NPHS1* and *NPHS2* mutations has been reported in CNS patients, but the clinical significance of this is not clear [11, 19].

No systematic analysis of clinical findings in CNS patients with *NPHS2* mutations has been published. The severity of proteinuria and, thus, the clinical findings are more variable than in NPHS1 patients [16]. The kidney histology often, but not exclusively, shows focal and segmental glomerular sclerosis (FSGS). Patients develop end-stage renal disease (ESRD) at the age of a few years [17]. Podocin is only expressed in kidney glomerulus, and no major extrarenal manifestations are present. As is the case in NPHS1, minor cardiac problems have been reported.

### Other genetic forms

Wilms’ tumor suppressor gene (*WT1*) encodes for a transcription factor WT1, which plays a crucial role in the embryonic development of the kidney and genitalia (Fig. 1). It is abundantly expressed in podocytes and controls cellular functions, such as nephrin expression. Mutations in *WT1* may cause several types of developmental syndromes (Denys-Drash, Frasier, and WAGR syndromes) manifesting in childhood [20–22]. *WT1* mutations can also cause an isolated kidney disease, with NS appearing in the first 3 months of life [17, 23]. They account for a few percent of CNS cases. Patients may have moderate proteinuria, and renal biopsy most often reveals diffuse mesangial sclerosis (DMS) of glomeruli.

A new genetic entity comprising CNS and distinct ocular anomalies with microcoria as the leading clinical feature (Pierson syndrome) was described in 2004 [24]. The disorder is caused by mutations in the laminin-β2 gene (*LAMB2*). Laminin-β2 is a component of GBM, where it is crucial for the network structure and anchoring of GBM to podocyte foot processes (Fig. 1). Interestingly, this was the first clear evidence that genetic defects in the GBM components can cause CNS in humans. Later studies revealed that the spectrum of *LAMB2*-associated disorders is broader than first suggested, and CNS patients without eye involvement have been described [25].

Galloway-Mowat syndrome (GMS) is characterized by NS with central nervous system anomalies, including microcephaly, psychomotor retardation, and brain anomalies [26]. Other extrarenal disorders, such as hiatus hernia, dysmorphic features, shortness, and diaphragmatic defects, have also been reported [2]. NS appears usually at the age of a few months (0–34 months), and kidney biopsy may show only minor changes [minor-change nephrotic syndrome (MCNS)], FSGS, or DMS. GMS is an autosomal recessive disorder, but the genetic defect(s) is still unknown. Podocytes resemble neuronal cells and share many structural proteins. Thus, genetic syndromes affecting both kidney and central nervous system are not surprising.

In addition to GMS, there are reports on other, unique, combinations of NS and extrarenal defects, including CNS-associated mitochondrial cytopathy [27], nail-patella syndrome [28], congenital disorder of glycosylation type I [29], Herlitz junctional epidermolysis bullosa [30], and CNS caused by mutations in the phospholipase C epsilon gene (*PLCE1*) [31].

### Nongenetic forms

Genetic defects account for the great majority of CNS cases, but especially in developing countries, infections are a possible etiology. Congenital syphilis has long been known to cause nephritic or nephrotic syndrome in the newborn [32]. Proteinuria and hematuria are present, but severe NS is less common. Kidney biopsy shows membranous nephropathy. Antimicrobial therapy with penicillin is curative provided that irreversible renal lesions have not developed. Toxoplasmosis, congenital rubella, and hepatitis B virus infection may also cause CNS. HIV can also infect kidney (podocytes) and is associated with nephropathy, including NS. It usually appears in children older than 1 year, but affected infants have been reported. An association of neonatal cytomegalovirus infection (CMV) and CNS has also been reported [33]. CMV infection is common during the first weeks of life, and detection of this virus in an infant with NS does not exclude an underlining genetic defect. This should be searched especially if ganciclovir therapy is not helpful. In addition to infections, CNS has been associated with maternal systemic lupus erythematosus and more recently with neonatal alloimmunization against neutral endopeptidase present on podocytes [34].

## Diagnosis of CNS

In severe forms of CNS, generalized edema, urinary protein > 20 g/L, and serum albumin level <10 g/L can be detected in the newborn period. The amount of proteinuria, however, varies in different entities, and the clinical signs may not be evident during the first weeks of life. Also, the true magnitude of proteinuria may be detectable only after partial correction of hypoproteinemia by albumin infusions. Small amounts of red blood cells and leucocytes are often present in urine. Serum creatinine and urea levels are variable. Renal function remains quite normal for the first months in NPHS1, but in other forms, kidney failure may develop faster. Blood pressure values can be low due to hypoproteinemia or elevated if renal failure is already present.

In newborns, the placental weight >25% of birth weight is present in NPHS1 but may be seen in other forms of CNS [14]. The kidneys may be of normal size or larger than normal in ultrasound scanning, and the renal cortex is often hyperechogenic. Search for possible nonrenal malformations is important, especially since they may give clues to the etiologic diagnosis. These include genital abnormalities (WT1), eye defects (LAMB2), and neurological disorders (Mowat–Galloway). Cardiac evaluation often reveals ventricular hypertrophy but no structural defects.

Renal biopsy does not reveal the etiology of CNS. As pointed out, the genetic defects may cause several types of glomerular lesions, such as mesangial expansion, FSGS, MCNS, and DMS, and the findings overlap in different entities. Also, the nonglomerular findings, such as tubular dilatations and interstitial fibrosis and inflammation, can be seen in all forms of proteinuric diseases. Thus, the indications for renal biopsy are not quite clear. The knowledge of severity of glomerular sclerosis and interstitial fibrosis may help in the assessment of treatment strategies. On the other hand, the lesions are focal, and the biopsy findings may be misleading. If immunohistochemistry for nephrin and podocin is available, analysis of their expression in a biopsy sample is useful. Total lack of either protein speaks for a severe disorder not responding to antiproteinuric therapy.

Genetic analysis is the method of choice for precise CNS diagnosis. The knowledge of etiology helps in assessing management and prognosis, in follow-up for possible associated symptoms, and in genetic counseling of the family. Analysis of *NPHS1* and *NPHS2* mutations is warranted in all CNS patients. These analyses are commercially available in Athena Diagnostics (www.athenadiagnostics.com). If no mutations are detected in these genes or if clinical findings speak for mutations in *WT1* or *LAMB2* gene, analysis of these genes can be obtained at research laboratories.

Prenatal diagnosis in families with a known risk for CNS should be based on genetic testing whenever possible. The results can be obtained fast if the mutations are known in advance. In case of no family history or if the mutations in the affected child were not identified, prenatal genetic testing is a challenge, since sequencing the *NPHS1* (29 exons) and *NPHS2* (eight exons) genes is time consuming and usually not possible within the short time frame available. NPHS1 especially can still be suspected prenatally based on elevated alpha-fetoprotein (AFP) levels in maternal serum and amniotic fluid. If the AFP concentration in amniotic fluid is very high and the ultrasound examination does not reveal fetal anencephaly or other malformations, NPHS1 is a probable diagnosis. However, heterozygous fetal carriers of *NPHS1* gene mutations may have temporarily elevated AFP levels in amniotic fluid and maternal serum, and repeated measurement of amniotic fluid AFP before the 20th week of pregnancy is recommended in cases with high AFP levels [35].

## CNS management

In contrast to most cases of childhood NS, therapy with steroids or other immunosuppressive drugs does not bring CNS into remission. The goals of therapy during the first months are to control edema and possible uremia, prevent and treat complications such as infections and thromboses, and provide optimal nutrition so that the child grows and develops as normally as possible (Table 2). In most cases, kidney transplantation is the only curative treatment.

**Table 2:** CNS management of infants with heavy proteinuria

Parameters in management of CNS
Protein substitution parenterally
20% albumin infusions (3–4 g/kg per day of albumin)
Nutrition
Hypercaloric diet (130 kcal/kg per day)
Protein supplementation (4 g/kg per day)
Lipid supplementation (rapeseed/sun flower oil)
A, D, E and water soluble vitamins
Calcium and magnesium supplementation
Medication
Antiproteinuric drugs (ACE-inhibitor, indomethacin)
Thyroxin supplementation
Anticoagulation (warfarin, aspirin, ATIII-infusion)
Parenteral antibiotics when bacterial infection suspected

### Albumin infusions

The magnitude of the protein losses into urine is crucial for therapeutic decision making. Heavy and constant proteinuria (10–100 gr/L) inevitably leads to life-threatening edema, protein malnutrition, reduced growth, and secondary complications. In these cases, protein substitution by parenteral albumin infusions is mandatory. Our practice in treating NPHS1 patients is to infuse 20% albumin solution together with a bolus of intravenous furosemide (0.5 mg/kg) using central venous catheters. The substitution is first divided into three 2-h infusions (starting dose 1–5 ml/kg per infusion) and after a few weeks given as one 6-h infusion during the night (up to 15–20 ml/kg each night; 3–4 g/kg of albumin). This substitution corrects hypoproteinemia only temporarily, but the patients do not have substantial edema.

### Medications

A reduction in the protein excretion using angiotensin-converting enzyme (ACE) inhibitors and indomethacin may be obtained in some infants with CNS [36]. Patients with severe *NPHS1* or *NPHS2* mutations inhibiting nephrin and podocin expression (stop codons, deletions, missense mutations) do not respond to this therapy, but in other cases, treatment with “antiproteinuric” medication is worth trying.

Due to the protein excretion, patients with NS often have low levels of serum thyroid-binding globulin and thyroxine. Thyroid-stimulating hormone (TSH) may be normal in the beginning but typically increases during the first months. Thus, thyroxine substitution is recommended in CNS patients. The medication can be started with 6.25–12.5 μg/day, and the dose can be adjusted according to TSH levels. Urinary protein losses result in imbalance of plasma coagulation factor levels, contributing to hypercoagubility and risk for thromboses. Thus, the use of aspirin and dipyridamole therapy has been recommended. Finnish NPHS1 patients are successfully treated with sodium warfarin from 3–4 weeks of age. Before surgical or vascular procedures, warfarin is stopped, and antithrombin III (50 IU/kg) is given to temporarily correct the deficiency.

Because of urinary losses of gamma globulin and complement factors and the use of indwelling catheters, nephrotic infants are prone to bacterial infections. Prophylactic use of antibiotics has been recommended, but in our experience, it is not helpful and only induces resistant bacterial strains. Similarly, prophylactic use of immunoglobulin infusion does not reduce the incidence of bacterial infections. On the other hand, a high degree of suspicion for septic infections is warranted. The symptoms are often vague and masked by signs of focal infections occurring at the same time. Parenteral antibiotic therapy should be started promptly on suspicion and should cover the major hospital strains of bacteria. Intravenous immunoglobulin is used along with the antibiotics in some centers. Response to treatment even in septic infection is usually excellent.

### Nutrition

Infants with severe CNS have traditionally been treated with a high-energy (130 kcal/kg per day) and a high-protein (3–4 g/kg per day) diet. Breast milk and milk formulas are first used, and the excess protein is given as a casein-based protein product. Glucose polymers are given to increase energy intake, and a mixture of rapeseed and sunflower oil is given to balance lipid levels [2]. The children also receive vitamin D_2_ (400 IU/day), which is changed to alpha-calcidiol when an increase of parathyroid hormone level is noticed. Multivitamin preparations are given according to the recommended dietary allowances for healthy children of the same age. Supplementary magnesium (50 mg/day) and calcium (500–1,000 mg/day) are also given to keep serum levels within the normal range. Daily water intake is 100–130 mL/kg. Most patients need a nasogastric tube to guarantee their energy intake.

### Nephrectomy

Some centers have a routine of performing unilateral nephrectomy to reduce protein losses [37]. This may decrease the frequency of the albumin infusions and help in the everyday management, so that renal transplantation can be postponed to an older age. Another approach is to perform an early bilateral nephrectomy and start peritoneal dialysis to avoid the complications encountered during the nephrotic stage. Our practice in treating NPHS1 patients is to perform bilateral nephrectomy when the child weighs about 7 kg and start peritoneal dialysis, which allows the infant stay at home. Renal transplantation is then performed a few months later when the child weighs more than 9 kg and the extraperitoneal placement of the graft is possible. The third possibility is to perform an early, preemptive renal transplantation with an intraperitoneal placement of the kidney graft. In this case, the nephrotic kidneys are removed at the same operation.

### Kidney transplantation

Renal transplantation has become an established mode of therapy for most children with CNS. The fact that CNS children are often transplanted at 1–2 years of age using adult-size kidneys may sometimes be surgically demanding and increase the risk for thrombotic and ureteral complications compared with older recipients. Postoperatively, abundant hydration of the recipient (3,000 ml/m^2^) is necessary to maintain optimum aortic and renal artery blood flow and avoid low-flow states that could damage the graft [38]. The use of immunosuppressive medication should be balanced in order to prevent rejection episodes, which may be clinically subtle, and on the other hand, avoid the many side effects associated with these drugs. Recurrence of NS in the graft is rare but has occurred in some NPHS1 children who developed antinephrin antibodies after transplantation. Treatment of the recurrence with cyclophosphamide and plasmapheresis often leads to remission [39].

Overall, the results of kidney transplantation in CNS are quite good and similar to those obtained in other etiologies. Patient survival at 5 years is over 90% and graft survival over 80% in registry databases and in single centers [40, 41]. Chronic allograft nephropathy, however, is a major problem also in these patients, and a second transplantation is inevitable when the patients become young adults.

## Conclusions

During the past few years, our knowledge on the genetic and molecular basis of CNS has greatly increased. Podocyte proteins play an important role in glomerular sieving, and mutations in genes encoding for nephrin, podocin, WT1 and lamininβ2 account for most cases of CNS. It is to be expected that more genetic defects will be found in CNS patients in the near future. Also, management of these infants has improved so that the outcome of CNS patients is very similar to other pediatric kidney patients requiring renal transplantation.

## **Questions** (Answers appear following the reference list)

1. The cardinal findings in CNS are:
  - Heavy proteinuria, high serum creatinine, and low serum cholesterol
  - Heavy proteinuria, low serum albumin, and generalized edema
  - Macroscopic hematuria, heavy proteinuria, high blood pressure values
2. The two most important structures preventing proteinuria in the kidney are:
  - Glomerular endothelial cells and basement membrane
  - Glomerular endothelial and epithelial cells
  - Podocyte slit diaphragm and glomerular basement membrane
3. The two most important genes associated with CNS are:
  - Nephrin (*NPHS1*) and podocin (*NPHS2*) genes
  - *WTI* and *LAMB2* genes
  - *CD2AP* and *NEPH1* genes
4. CNS infants with heavy proteinuria have a risk for:
  - Bleeding episodes and hypertensive crisis
  - Thrombotic and septic episodes
  - Hyperthyroidism and hypermagnesemia
5. The effective treatment in most cases of CNS is:
  - Prednisone and other immunosuppressants
  - Calcium blockers and indomethacin
  - Renal transplantation
